# Community stakeholder‐driven technology solutions towards rural health equity: A concept mapping study in Western Canada

**DOI:** 10.1111/hex.13627

**Published:** 2022-10-17

**Authors:** Cherisse L. Seaton, Pierre Rondier, Kathy L. Rush, Eric P. H. Li, Katrina Plamondon, Barb Pesut, Nelly D. Oelke, Sarah Dow‐Fleisner, Khalad Hasan, Leanne M. Currie, Donna Kurtz, Charlotte Jones, Joan L. Bottorff

**Affiliations:** ^1^ School of Nursing University of British Columbia, Okanagan Kelowna British Columbia Canada; ^2^ Research and Innovation Office University of British Columbia, Okanagan Kelowna British Columbia Canada; ^3^ Faculty of Management University of British Columbia, Okanagan Kelowna British Columbia Canada; ^4^ University of British Columbia, Okanagan Kelowna British Columbia Canada; ^5^ Rural Coordination Centre of British Columbia University of British Columbia, Okanagan Kelowna British Columbia Canada; ^6^ School of Social Work University of British Columbia, Okanagan Kelowna British Columbia Canada; ^7^ Computer Science University of British Columbia, Okanagan Kelowna British Columbia Canada; ^8^ School of Nursing University of British Columbia Vancouver British Columbia Canada; ^9^ Faculty of Medicine University of British Columbia, Okanagan Kelowna British Columbia Canada

**Keywords:** community‐based research, concept mapping, equity, health, participatory research, rural, technology

## Abstract

**Background:**

Technology holds great potential for promoting health equity for rural populations, who have more chronic illnesses than their urban counterparts but less access to services. Yet, more participatory research approaches are needed to gather community‐driven health technology solutions. The purpose was to collaboratively identify and prioritize action strategies for using technology to promote rural health equity through community stakeholder engagement.

**Methods:**

Concept mapping, a quantitative statistical technique, embedded within a qualitative approach, was used to identify and integrate technological solutions towards rural health equity from community stakeholders in three steps: (1) idea generation; (2) sorting and rating feasibility/importance and (3) group interpretation. Purposeful recruitment strategies were used to recruit key stakeholders and organizational representatives from targeted rural communities.

**Results:**

Overall, 34 rural community stakeholders from western Canada (76% female, mean age = 55.4 years) participated in the concept mapping process. In Step 1, 84 ideas were generated that were reduced to a pool of 30. Multidimensional scaling and cluster analysis resulted in a six‐cluster map representing how technological solutions can contribute toward rural health equity. The clusters of ideas included technological solutions and applications, but also ideas to make health care more accessible regardless of location, training and support in the use of technology, ensuring digital tools are simplified for ease of use, technologies to support collaboration among healthcare professionals and ideas for overcoming challenges to data sharing across health systems/networks. Each cluster included ideas that were rated as equally important and feasible. Key themes included organizational and individual‐level solutions and connecting patients to newly developed technologies.

**Conclusions:**

Overall, the grouping of solutions revealed that technological applications require not only access but also support and collaboration. Concept mapping is a tool that can engage rural community stakeholders in the identification of technological solutions for promoting rural health equity.

**Patient or Public Contribution:**

Rural community stakeholders were involved in the generation and interpretation of technological solutions towards rural health equity in a three‐step process: (1) individual brainstorming of ideas, (2) sorting and rating all ideas generated and (3) collective interpretation and group consensus on final results.

## 1 BACKGROUND

Health inequities are systemic and avoidable differences in health that are caused by the unfair distribution of resources, wealth and power in society.^1^ People who experience systemic disadvantages (e.g., due to racism, exclusion, impacts of colonization, socioeconomic status or access to services) also experience greater burdens of health inequities.^2^, ^3^ Social and structural determinants of health that contribute to rural health inequities include financial, social and geographical difficulties such as travel to access care; reduced access to healthcare professionals; lack of healthcare facilities, services and equipment; inadequate infrastructure and lack of rural‐specific programmes.^4^, ^5^ In western Canada, dispersed rural and remote community settings accentuate the drivers of ill health and limit access to health care.^5^, ^6^ Chronic diseases such as cardiovascular disease, asthma and diabetes along with poor mental health, obesity, lower life expectancy and potentially avoidable mortality are higher in rural and remote areas than in urban areas of Canada.^7^ Living with chronic illness can increase healthcare needs which can exacerbate the drivers of health inequity. One possible solution for reducing health inequities is the use of technology to promote inclusive health and social care for disadvantaged rural populations. Research examining the ways technology solutions can best integrate rural needs, values and strengths can therefore be an important contributor to advancing rural health equity.

Digital health technologies are revolutionizing health and social care, opening new possibilities for increasing access, reducing inequities and promoting equity. For example, mobile technologies such as mobile phone apps hold considerable potential to reduce inequities because of their extensive use across all social groups.^8^ Remote monitoring and synchronous video‐based technologies offer opportunities to develop community‐based interventions and reduce the need for proximity or travel to healthcare providers.^9^ Virtual care (including telehealth), defined as any remote interaction between patients and their circle of care using communication/information technology,^10^ rapidly expanded to rural and remote communities during the COVID‐19 pandemic to provide services previously unavailable.^11^, ^12^ Although digital access is far from equal across geographic contexts, and many rural communities lack a digital infrastructure,^13^, ^14^ this landscape is shifting. Just before COVID‐19, one provincial government in Canada invested in the development of a Digital Health Strategy and funding for high‐speed internet to 200 rural and Indigenous communities, enhancing opportunities for harnessing technological solutions for more equitable access to health‐related resources, information and services.^15^, ^16^ This expanded infrastructure creates opportunities to move beyond urban contexts to customize technological solutions to rural locales.

Yet, geographical place shapes how technology is used or not used,^17^ and the rural context is diverse and an especially important consideration with the expedited need for technology across jurisdictions as a result of COVID‐19. Participatory community‐driven research is ideally suited to identify acceptable and relevant user‐driven solutions to pragmatic real‐life issues.^18^, ^19^ Community‐engaged research is a people‐centred approach building from community strengths and priorities. It is crucial to promoting health equity by integrating diverse voices including those of marginalized groups^20^, ^21^ and aligned with the direction of this study. Involving communities as partners in the research process reduces the power differential that often characterizes a top‐down approach and enhances the planning, conduct and usability of the research.^22^, ^23^ Engaging rural community stakeholders in the co‐identification and co‐creation of community‐centred solutions also facilitate integrated knowledge translation.^24^ In the current study, a knowledge‐to‐action technique, known as concept mapping, was used to engage rural community stakeholders, giving them an active voice in generating solutions and bringing their community, experiential, professional and tacit knowledge into shaping a collective understanding of how technology relates to rural health equity. The research question guiding the study was: What are priority technology solutions to support the health and well‐being of people living with chronic illness in rural communities?

## 2 METHODS

### 2.1 Study design

A concept mapping approach was used to generate and compile technology solutions for rural community members living with chronic illness. Concept mapping is well suited to identify future strategic planning and evaluation^25^, ^26^ and to explore lived experience in participatory public health research.^18^ Concept mapping allows for both individual brainstorming of ideas as well as mapping complex concepts to reveal an underlying structure not directly identified by individual participants.^27^, ^28^ The concept mapping approach used in this study was informed by the work of Trochim^26^ and Burke et al.^18^ in that quantitative statistical methods, used to synthesize and map participants' solutions to a complex problem, were combined with group discussion and consensus on the final mapped solution. Harmonized ethics approval was received from the University of British Columbia Research Ethics Board (#H20‐00075), the Interior Health Research Ethics Board (2019‐20‐094‐H) and the BC Emergency Health Services (BCEHS) and the Research and Evaluation Subcommittee (File #: 51500‐01).

### 2.2 Study setting and recruitment

This study was conducted in a western region of Canada that is characterized by substantial geographic differences in urban and rural characteristics, with over 40% of the population living outside two major metropolitan areas.^29^ The geography is diverse (e.g., forests, lakes, deserts, grass plains) and 75% of the region is covered by mountains.^30^ In contrast to urban areas, demographic ageing data indicate a larger proportion of older adults in rural communities.^31^ Ten rural communities (populations ranging from 957 to just over 10,000) were targeted in the interior region of the province (where medical services were provided through one of the provincial health regions) to leverage research team connections. The rural communities varied with respect to Statistics Canada's Index of Remoteness (based on population and travel to the nearest population centre) as five communities were classified as ‘less accessible areas’, and five were ‘remote areas’.^32^ With respect to broadband infrastructure, the represented communities all had either 50/10 Mbps (*n* = 7) or 25/5 Mbps (*n* = 3) available at the community centre according to the National Broadband Internet Service Map.^33^

A purposeful recruitment method using emails, advertisements and the snowball method was used to invite rural‐living community advocates, health service providers and those living with chronic disease or caring for someone with chronic disease. Participants who were 19 years or older and either lived or worked in or near a rural community in British Columbia (BC) or had relevant expertise about technology or local organizations that might support rural health were invited to participate.

### 2.3 Data collection

**Figure 1:**
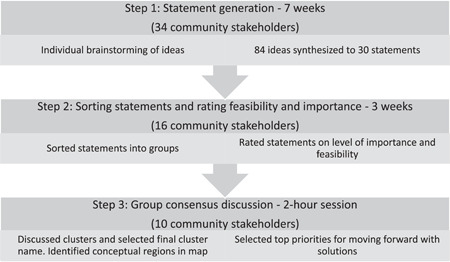
Flow diagram of the concept mapping process

Polygon Research Inc., a Canadian company based in Quebec, provided the concept mapping platform, Insight Forming,^34^ which was used to facilitate the collection, processing and visualization of data. Participants were sent a link to the secure platform, where they entered an email address to create an account and ‘login’ to the study to complete the online questionnaire and consent forms. The online, asynchronous concept mapping process allowed us to reach a broad group of stakeholders, circumventing geographical limitations, though one participant completed a paper version that was entered by the research staff. In the present study, our goal was to collaborate with our community partners in mapping the final solution; thus, we also organized a virtual group discussion session for participants to co‐interpret a visual solution. The online concept mapping process included three steps: Step 1: Generate Statements; Step 2a: Sort Statements; Step 2b: Importance and Feasibility Rating; Step 3: Consensus Discussion (see Figure 1 for a flow diagram of the concept mapping process and timeline). All participants were also asked to complete a short (5 min) demographic questionnaire. This included age; gender, marital status, sector/affiliation (e.g., health/social services, nonprofit/charitable organization, education, policy/government); education level; ethnicity; status as living with, or caring for someone with, chronic illness; access to the internet at home; adequacy of internet access (reliability/quality) on a scale ranging from poor (1) to excellent (10) and community name. Participants were provided with a $10 eGift card for participating in Step 1, a $20 eGift card for participating in Step 2 and a $50 eGift card for participating in Step 3.

#### 2.3.1 Step 1: Generate Statements

In Step 1, participants completed an idea generation activity between 14 July and 3 September 2020 (7 weeks) in response to the question: ‘What are possible technology solutions that could address the health and well‐being issues of people living with chronic illness in rural BC communities?’ The number of responses participants could generate was not limited in this first step. Once enough stakeholders had completed the first step to start seeing saturation in the responses, complex responses were broken down by two study authors (C. L. S. and P. R.) into individual ideas, duplicate responses were collapsed, language was simplified to ensure understanding and ultimately responses reflecting similar content were synthesized into global statements.

#### 2.3.2 Step 2a: Sort Statements

In Step 2a, participants were invited to individually sort the final pool of synthesized statements generated by all participants into groups of conceptually similar ideas and provide names for these groups over a 3‐week period (10–30 October 2020). Instructions told participants: ‘In this step you are being asked to sort the items into groups. You can sort in whatever way makes sense to you, and give your groups any name you would like. Please try to sort all items; however, each group you create needs to contain at least 2 items’. Participants could create as many groups to represent the statements as they saw fit.

#### 2.3.3 Step 2b: Importance and Feasibility Rating

Simultaneously, in Step 2b, participants rated each of the synthesized statements in terms of importance and feasibility. Participants were asked to rate as they felt best: ‘What level of importance do you consider this statement?’ (from 1 = not important at all to 6 = very important) and ‘What would be the feasibility of this solution for rural BC communities?’ (from 1 = very low to 6 = very high).

#### 2.3.4 Step 3: Group Consensus Discussion

In Step 3, a 2‐h virtual discussion session (4 November 2020) was held using Zoom where the clusters were presented visually and participants discussed the individual solutions in each cluster and their association. Options for cluster names (based on names participants had given groups when sorting) were presented in a poll to participants. The top choices for names were discussed and often modified from what was originally presented in the poll. After clusters were named, participants were asked in another poll: ‘Which cluster is your top priority for action’. Finally, participants were asked to discuss the overall organization of the clusters of ideas and to collectively identify the conceptual regions or ‘bigger picture’ that could be present in the map of solutions.

### 2.4 Data analysis

Hierarchical cluster analysis and nonmetric multidimensional scaling (nMDS) were used to merge the sorted items into a combined set of clusters.^35^ The concept map visually represented these concepts in two‐dimensional space. A Kruskal stress index was computed to assess the goodness of fit.^36^

Two hierarchical clustering methods, the unweighted pair‐group method using arithmetic averages (UPGMA) and Ward's 2 minimum variance clustering, were compared to assess the reliability of the cluster solution.^37^, ^38^ These two solutions were compared on a number of indices. For example, Cophenetic correlations (Spearman's *ρ* and Kendall's *τ*) and dendrograms (alignment quality and Baker's *γ* correlation) were compared for UPGAMA and Ward's clustering. An iterative process was used to determine the best‐fitting solution and the final number of clusters. Silhouette index scores aided the determination of whether individual items belonged in their cluster; positive scores indicate belonging to a cluster (and the higher the silhouette score, the more central the item is to its cluster), null values indicate an item is between two clusters, and negative values indicate an item is closer to another cluster.^39^ Finally, cluster stability was assessed by computing the average Jaccard similarities between bootstrapped pairwise clustering solutions.^40^ Jaccard similarity coefficient values range from 0 (no relationship) to 1 (perfect relationship) with higher values indicating more stable clusters.

Descriptive statistics were used to summarize the demographic data in SPSS version 27.^41^ Mean ratings of feasibility and importance for each technological solution were generated by taking the average of all participant ratings. Then a scatterplot of mean ratings of importance and feasibility was used to generate a ‘go‐zone’. In concept mapping, a ‘go‐zone’ graph is used to identify items that are rated both highly important and highly feasible.^28^ A ranking of average ratings (means) was used to determine cutoff points for the ‘go‐zone’.

The group consensus discussion was audio‐recorded and transcribed. Using a content analysis approach,^42^ participants' interpretations of the clusters and main areas of consensus were identified, and representative quotes were selected to illustrate participants' perceptions.

## 3 RESULTS

### 3.1 Participants and sample characteristics

A total of 34 people (26 females, 7 males and 1 preferred not to answer) participated in this concept mapping process. Most participants (*n* = 30; 88.2%) identified as residents of rural communities in the study region, and 4 (11.8%) were stakeholders with technology or rural health backgrounds from larger urban centres. There were one to seven participants from each community (median = 3). All 34 (100%) participants reported having access to the internet at home, and adequacy of internet access (reliability/quality) was rated good/excellent (7, 8, 9 or 10) for 31 (91.2%) participants; however, 3 (8.8%) participants rated their internet quality lower (one 4, one 5, one 6). Participant's ages ranged from 26 to 90 years (mean = 55.41 years; SD = 14.82; median = 56.50). The majority of participants were married (*n* = 28; 82.4%), and 24 (70.6%) had a university degree. Five (14.7%) participants identified as First Nations, Metis or Inuit, 28 (82.4%) identified as Caucasian and one participant (2.9%) preferred to not answer. Participants were affiliated with multiple sectors: 68% self‐identified as being in the health and/or social services sector, 44% in nonprofit/charitable organizations, 41% in education and 38% in policy/government. Almost half of the participants also were either living with a chronic illness (20%) or were caring for someone living with a chronic illness (25%). Common types of chronic illness participants reported included asthma, arthritis and diabetes; types of chronic illnesses participants reported caring for in another were varied and included cardiovascular conditions, chronic pain and mental health concerns.

### 3.2 Idea generation, sorting and rating

**Figure 2:**
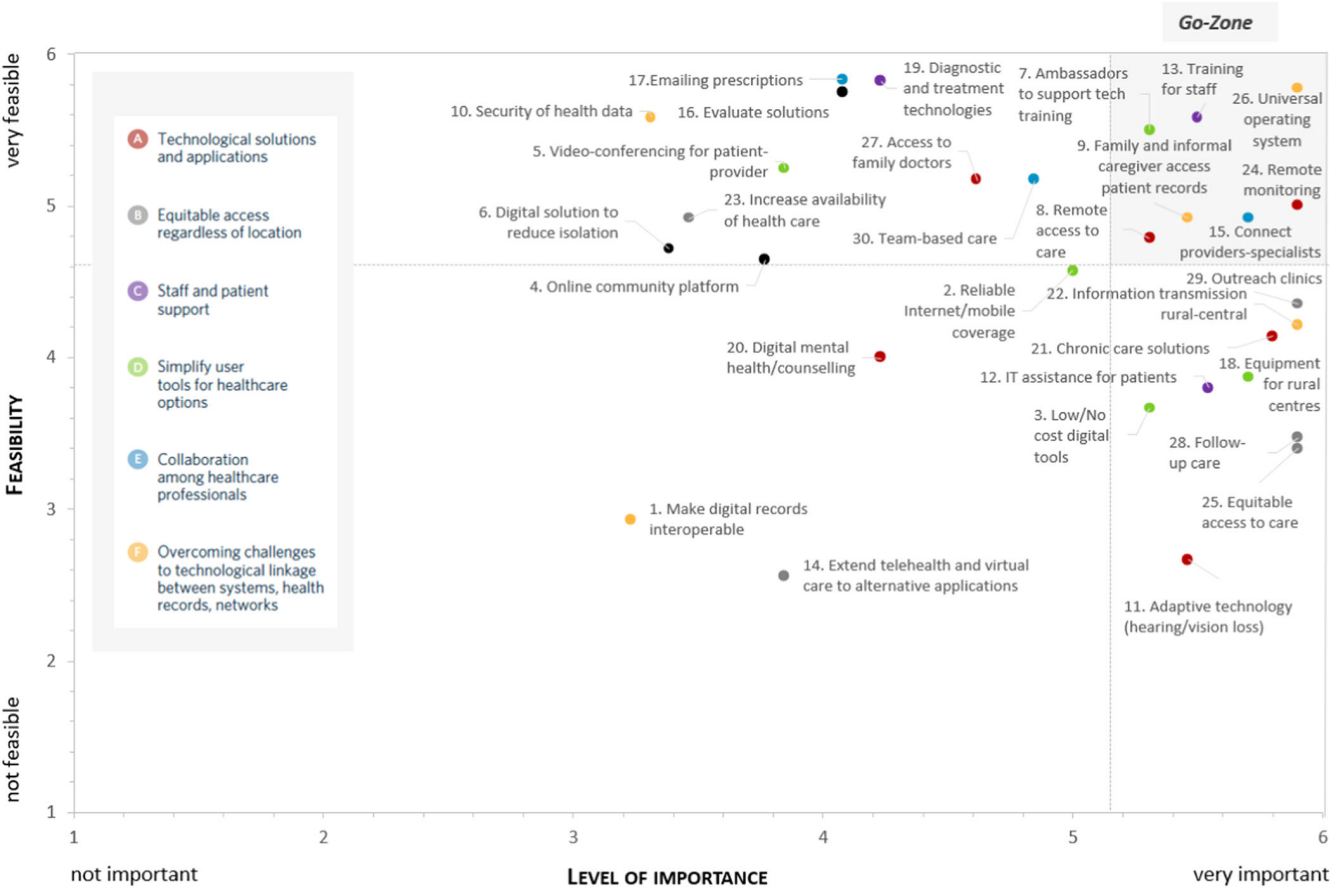
Statements by participant ratings of importance and feasibility including a ‘go‐zone’ (top right) of items rated both highly important and highly feasible. In black are three items that were not part of the final six‐cluster solution.

In Step 1, participants generated a total of 84 initial ideas. The three most commonly recurring items surrounded ensuring video conferencing is available for meaningful patient–provider interactions (mentioned 13 times), access to affordable, high‐quality internet and cellular coverage (mentioned 10 times) and providing ambassadors to support patients and families with training in the use of technology (mentioned 8 times). The 84 ideas were reduced by the study researchers (as duplicate ideas were combined) into 30 representative statements for sorting and rating (see 30 statements in Table 1). In Step 2, 16 participants (56% female; 87.5% rural; *M* age = 59 years; 81% married; 81% University degree; 75% Caucasian) returned and sorted the ideas into groups and then rated items in terms of their importance and feasibility. Figure 2 presents the ideas according to the average rating of importance and feasibility. All 30 ideas received average ratings of importance of 3 or higher. The highest‐rated statements in terms of both feasibility and importance are captured in the ‘go‐zone’ at the top right of Figure 2.

**Table 1:** Statements organized by cluster, including average ratings of importance (range 1–6) and feasibility (range 1–6)

Statement	Importance		Feasibility	
	Mean	Standard deviation	Mean	Standard deviation
CLUSTER A: Technological solutions and applications				
24. Develop smartphone applications for remote patient monitoring	5.9	0.3	5.0	0.7
21. Adopt technological solutions to meet supportive, chronic care needs in the community	5.8	0.4	4.1	0.9
11. Develop adaptive technology to address mobility or sensory needs (e.g., hearing impairment, vision loss) of patients connecting by phone	5.5	0.8	2.7	1.0
8. Develop technological solutions for remote and virtual meetings when access to care is difficult (weather, travel distance)	5.3	0.9	4.8	0.9
27. Develop technological solutions to help rural communities gain access to family doctors	4.6	1.0	5.2	0.8
20. Develop digital solutions for real‐time mental health care and counselling sessions	4.2	1.1	4.0	0.9
Average values	5.2	0.7	4.3	0.9
CLUSTER B: Equitable access regardless of location				
28. Ensure follow‐ups (e.g., via telephone/video) for patients who've seen a specialist but do not have a family physician	5.9	0.3	3.5	0.9
29. Provide basic services in outreach clinics in small communities using mobile technology	5.9	0.3	4.4	1.1
25. Ensure equitable access to high quality care regardless of location (e.g., home care vs on‐site care; rural/urban)	5.9	0.3	3.4	1.0
14. Make telehealth available in a variety of healthcare settings (e.g., acupuncture clinics)	3.8	0.8	2.6	1.2
23. Provide digital solutions that increase on‐demand availability of health care services (availability 24/7, shorten wait times)	3.5	0.9	4.9	0.8
Average values	5.0	0.5	3.7	1.0
CLUSTER C: Staff and patient support				
12. Increase access to free real‐time assistance by creating staffing position within health organization for technological support (e.g., an ‘IT’ department patients can connect with by phone or online chat)	5.5	0.7	3.8	0.9
13. Provide education/training and support (i.e., paid time) for rural staff to ensure they can use the technologies available to them	5.5	0.7	5.6	0.7
19. Adopt technologies to improve personalized diagnostic and treatment processes	4.2	1.1	5.8	0.6
Average values	5.1	0.8	5.1	0.7
CLUSTER D: Simplify user tools for healthcare options				
18. Increase rural health centre access to equipment (e.g., computers, satellite)	5.7	0.5	3.9	0.9
7. Provide ambassadors to support patients and families with training to use technology	5.3	0.9	5.5	0.7
3. Ensure digital tools are available to patients with new audio‐visual capabilities (high quality cameras + microphones) at low‐to‐no cost	5.3	0.9	3.7	1.0
2. Ensure access to reliable, affordable and high‐quality internet and cellular coverage	5.0	0.9	4.6	0.9
5. Ensure video conferencing (e.g., Zoom, Google, GoToMeeting, Skype) available for meaningful patient–provider interactions	3.8	0.8	5.3	0.9
Average values	5.0	0.8	4.6	0.9
CLUSTER E: Collaboration among healthcare professionals				
15. Connect local care providers with specialists in larger centres for continuity of patient care	5.7	0.5	4.9	0.8
30. Use technology to support team‐based care	4.8	1.0	5.2	0.8
17. Continue process of emailing prescriptions to pharmacists	4.1	1.0	5.8	0.6
Average values	4.9	0.8	5.3	0.7
CLUSTER F: Overcoming challenges to technological linkages between systems, health records, networks				
26. Ensure all the computer systems within all the health care system are using the same operating system (to improve the transmission of data between centres)	5.9	0.3	5.8	0.7
22. Adopt technological solutions to enhance transmission of information between rural and central health centres	5.9	0.3	4.2	1.0
9. Provide patients and caregivers/family members digital access to patient's health records to support care from a distance	5.5	0.8	4.9	0.9
10. Explore technological solutions to improve security of personal health data and allow patient to choose who can access	3.3	1.4	5.6	0.7
1. Digitize up to date health records that link patient's information (health conditions and status) across all service providers	3.2	0.9	2.9	1.0
Average values	4.8	0.7	4.7	0.8
Statements not part of any clusters (removed)				
16. Implement ongoing evaluation of technological solutions	4.1	1.0	5.8	0.6
4. Create a platform to support online community engagement for communication and planning (e.g., organizing ride share)	3.8	0.8	4.6	0.8
6. Use digital solutions to facilitate connecting with family, friends and support groups to reduce isolation and loneliness	3.4	0.9	4.7	0.9

### 3.3 Generating a concept map

Using hierarchical cluster analysis and nMDS, the items of individual participants sorted into groups were combined into a set of six clusters. Table 1 presents the abbreviated statements organized by cluster, along with average ratings of importance and feasibility. The Kruskal stress index for this two‐dimensional solution was 0.199. (Lower Kruskal stress index scores represent a better fit, with the following guidelines: Values approaching 0.3 suggest an arbitrary solution [cannot be interpreted], values above 0.2 must be interpreted with caution, values below 0.1 are considered fair, and values below 0.05 suggest a good fit.^36^ However, nMDS is a robust technique, and because our primary interest was to interpret the organization of concepts in clusters, moderate values of stress [i.e., values approaching 0.2] can be tolerated in this context^43^). Three items were removed. The item ‘Implement ongoing evaluation of technological solutions’ was removed because it was left unsorted by 6 of the 16 participants. The item ‘Create a platform to support online community engagement for communication and planning (e.g., organizing ride share)’ was removed because it had a negative silhouette value in the final solution, meaning it was closer to another cluster. The item ‘Use digital solutions to facilitate contact with family, friends and support groups to reduce isolation and loneliness’ was removed because it had a low internal validity for the clustering solutions, meaning most participants sorted it into differing groups.

**Figure 3:**
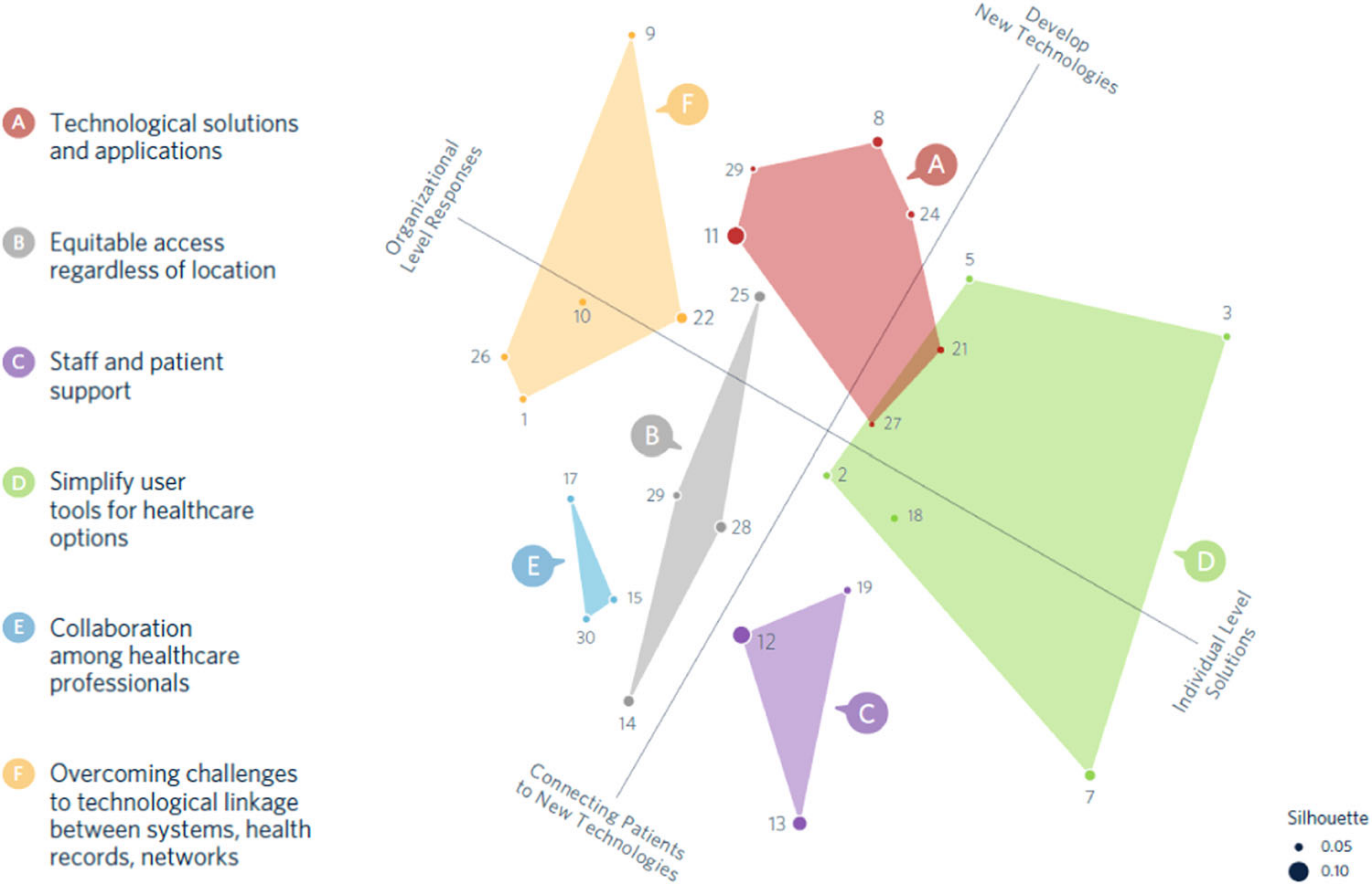
Concept map of 27 statements with a six‐cluster solution. Higher silhouette index scores suggest items are more central to their cluster.

The UPGMA and Ward's clustering methods provided highly similar results on all indices suggesting the six‐cluster solution was reliable. Ultimately, the UPGMA method was selected, as some indices (e.g., cluster stability) were slightly higher than using Ward's clustering. Using the UPGMA method, average Jaccard similarities between bootstrapped pairwise clustering solutions for the six clusters ranged from 0.61 to 0.82, suggesting at least somewhat valid, stable clusters when resampling the data. Figure 3 presents a concept map of the 27 statements with a six‐cluster solution.

### 3.4 Naming the clusters

In Step 3, 10 participants (70% female; 90% rural; *M* age = 59.6 years; 80% married; 80% University degree; 80% Caucasian) who completed Step 2 attended a 2‐h virtual session to collectively interpret the results, though 2 of these joined using audio‐only, given low internet quality for video.

The first named cluster was Technological Solutions and Applications (Cluster A) and it included smartphone applications and technology solutions. This cluster captured diverse technology applications to meet a range of needs for people with chronic illnesses in rural communities. Some were general applications such as for supportive, chronic care and some were specific applications to address mobility sensory needs, mental health, monitoring or giving access to primary care providers. Indeed, as one participant explained, ‘Technology could be many simple things as well. It could be manufacturing or med devices’ (P3). Overall, participants interpreted this cluster as being about ‘developing technological solutions to help rural communities gain access [to care]’ (P9) which otherwise may be too costly or difficult to access. As one female rural community resident explained: ‘Our tertiary hospital would be a good four‐hour drive—it's an overnight trip, which means then there's accommodation expenses as well. So in some cases, people just don't engage with those site visits’ (P1). The technology solutions in this cluster were seen as a way to create options for remote engagement with health care.

Equitable Access Regardless of Location (Cluster B) consisted of ideas that would make access to health care more equitable. These ranged from mobile technology to enable local community outreach clinics to do virtual visits to ensure remote patients without a family doctor are not ‘lost’ to follow‐up. Ideas in this cluster surrounded ensuring patients could access care in a variety of settings, including digital solutions for timely, on‐demand (24/7) care. One female rural community health sector worker explained: ‘Most of this is about availability of care in a variety of different contexts and making sure that even if people don't have certain basic things like a family physician, that they still have that available’ (P2).

Staff and Patient Support (Cluster C) involved training and support for both patients and healthcare providers so they can use the available technology. The larger meaning of this cluster for participants was access to real‐time assistance and training needed for any technology to be successfully deployed. As one female rural community member described:

One of the things that I've seen is that we've got this technology, but if one person wasn't there, the person that knows how to use it, then nobody gets to use the technology. It's been a concern for all of us because that shouldn't be that way. Right? If the technology is there, there has to be people that can access it for the patient. (P10)

Simplify User Tools for Healthcare (Cluster D), encompassed not only training for patients but also ensuring free or low‐cost digital tools and internet/mobile coverage were available, enabling people to access meaningful care. This cluster included a range of ideas related to ensuring digital tools and solutions are simplified so that patients can use them. As one male rural community participant explained:

Simple and reliable are not the same. You can reliably have a really complex system that nobody can use…. When you're the user, what you want is almost a manual button that you just push ‘start’ and it works. It's like your car, right? That's a very complex piece of equipment, but it's had user input of, ‘I don't want to go out and hook up a battery and do this and do that. And all the rest of it’. So these days you can just sit in your car with a thing [key fob] in your pocket and your car can start. And so it's part of designing the technology, not to suit the technology developers, but to suit the end user—simple access. (P6)

Collaboration among healthcare professionals (Cluster E), captured technologies for improving provider‐to‐provider interactions, along with using technology to support team‐based care. After a discussion that collaboration ‘involved the patient as well, because the patient always has to be at the center’ (P8), participants identified the importance of the patient being at the centre of team‐based care as largely missing from the map. One female rural community member explained:

Team‐based care is a really important aspect of what we've been talking about today. And so far, we don't have any cluster that really reflects that … Team‐based care is really the name of the game. It's why we want to do all of these technological solutions to access to healthcare. We want to expand the team. It's not just a physician and a person anymore. (P1)

Overcoming challenges to technology linkages between systems, health records, networks (Cluster F) encompassed interoperability between health system records and primary care provider electronic health records to improve quality of care. For example, linking patient health information across service providers and allowing patients and their caregivers access to digital records. One female rural community stakeholder explained: ‘they're trying to make interoperable networks to enable whoever wants to have[access to a record] go and use it’ (P3).

### 3.5 Interpreting the map

Clusters A and B were selected as the top priorities for moving forward with solutions. Participants noted that although items at the top right of the map were about the development of new technologies, items at the bottom left highlighted the importance of connecting patients to technology. The ‘human’ aspects of technology use, including ambassadors, training and support were seen as critical for ‘Connecting patients with new technologies’ (P10). According to one female rural community member:

And that's where I go back to the ambassador, to the actual person who knows what's going on, and can push the buttons, and can ensure that when I want to talk to the specialist, I can talk to the specialist and not worry about clicking and whatever else. (P1)

Organizational or team‐level ideas (e.g., use technology to support team‐based care) were grouped towards the top left of the map, whereas items related to specific tools or solutions (e.g., ensure digital tools are available to patients) were grouped to the bottom right of the map. Cluster B, named ‘equitable access regardless of location’, was at the centre of the map, with all other clusters surrounding it, suggesting these ideas are centrally related to all the others. ‘It's about ensuring access anywhere and everywhere’ (P1). In addition, the individual item ‘ensure access to reliable, affordable and high‐quality internet and cellular coverage’, part of Cluster D, was also near the centre of the map. Participants explained that network access was key to making technology solutions possible: ‘Good access to internet is a technological solution … it's a piece of it’ (P6).

## 4 DISCUSSION

The purpose of this study was to collaboratively identify and prioritize action strategies for using technology to promote rural health equity. With the engagement of diverse rural community stakeholders, the findings present a co‐created set of technology solutions to support the health and well‐being of people living with chronic illness in rural communities. Although the study results are based on experiences in rural settings in western Canada, the findings may also hold value for other rural contexts where similar factors influence health inequities.

Findings from this concept mapping study offer technology solutions to begin to redress well‐known rural inequities and unfair structural and social determinants of health. In addition to cost and travel time/distance, rural communities face additional difficulties travelling for health care, such as dangerous weather, mountainous terrain and the dependence on ferry services for island communities.^4^ The Technological Solutions and Applications (Cluster A), as well as Equitable Access Regardless of Location (Cluster B), clusters both include solutions for accessing care without travel. The shortage of healthcare professionals in rural communities has adverse consequences for rural‐living people, as they may miss the treatment or go through treatment and recovery outside of their community without the support of family and friends.^4^ Technology solutions were proposed for outreach clinics and to help rural communities gain access to primary care providers mirroring the suggestions from rural citizen‐patients in the recent Rural Evidence Review (2019). Our findings suggest that, from the perspective of rural community stakeholders, technology could be used so that living rurally in itself does not serve as a structural determinant of health.

Yet, in the current study participants also introduced Staff and Patient Support (Cluster C), and the need to Simplify User Tools for Healthcare Options (Cluster D) as essential for ensuring technology was accessible. Indeed, in the open‐ended feedback, the group did not place technology solutions as their only priority and did not see health technologies as a ‘one size fits all’ solution. The human aspect of technology was seen as critically important for ‘connecting’ patients with new technologies. Further, solutions in the Collaboration among Healthcare Professionals (Cluster E) and Overcoming Challenges to Technological Linkages (Cluster F) clusters suggest that technology is not necessarily seen as the main driver that will transform the health system equitably but an essential component that supports building connections between the various actors of the health system. Participants discussed challenges and risks more than the opportunities these technologies represent. They emphasized collaboration, training and human support in addition to the technology solutions themselves. Exposing both the potential and pitfalls of technology to address rural health equity reflects the value of community‐engaged research in promoting a balance of perspectives and richness/diversity in the generation of solutions not possible with a top‐down approach.^22^, ^23^ Indeed, all of the ideas were rated as highly important, reflecting the complex inter‐related challenges often faced by rural communities and the need for multilevel solutions in underserved rural populations to address the lack of equitable access to health.^5^, ^44^ Ensuring access to reliable, affordable and high‐quality internet and cellular coverage was not only at the centre of the concept map, but it was also one of the most frequently occurring suggestions in the original pool of 84 ideas. This finding, in part, reflects the fact that in Canada, although 97% of citizens living in urban regions have access to high‐speed internet, only 46% of citizens living in rural communities have access to the same service.^45^ Adequate digital infrastructure is imperative for rural communities to engage in every area of life and is key to reducing inequities experienced by people living in rural communities.

Another commonly recurring suggestion surrounded technology solutions for patient–provider interactions, possibly reflecting the pressing human resource shortages in rural communities.^44^ Yet, virtual care used to its full capacity (e.g., video visits) requires adequate broadband access, which is often limited in rural and underserved settings.^46^ Indeed, a previous systematic review suggested that videoconferencing improved the accuracy of diagnoses and reduced readmission rates compared to the telephone.^47^ If technological solutions are to effectively begin addressing rural healthcare challenges,^5^ the necessary technology infrastructure to support high‐quality care will need to be in place.

It is notable that the highest‐rated individual ideas in terms of both importance and feasibility (captured in a ‘go‐zone’ in Figure 2) included statements from five of the six clusters. Of the six items captured in the ‘go‐zone’, three related to developing digital solutions, but three other solutions, linked indirectly to the development of technology solutions, emphasized selecting, developing, using and evaluating technology solutions while placing the patient and health practitioners at the centre. Providing ambassadors to support training to use technology was among the most frequently occurring suggestion in the original pool of 84 ideas, reinforcing interest in the ‘human’ support for multiple rural community stakeholders.

An emphasis on digital skills training should be an essential component in the introduction of any new technology. In rural locales, in particular, there may be a strong preference for face‐to‐face training,^48^ consistent with the present findings. Yet digital literacy, defined as the ability to use communication and information technologies to access, evaluate and communicate information^49^ is often overlooked in the development of technology‐based interventions, limiting accessibility.^50^ Indeed, higher digital literacy was related to higher satisfaction with telemedicine in a recent study of rural community telemedicine use during COVID‐19.^51^ Education and training could be tailored to local needs, preferences and skills of healthcare teams and communities to ensure new technologies can be successfully adopted. In order for technology to contribute to advancing equity in rural communities, accessibility considerations encompass hardware, connectivity (cellular and internet service at adequate bandwidth) and informational technology supports and skills.

Importantly, rural voices must be included in the design and delivery of equity‐advancing use of technology. The present findings reinforce the need for participatory research to ensure acceptable user‐driven solutions are identified.^18^ In a review of 103 manuscripts that included concept mapping methodology, 38% employed high community engagement, with notable benefits such as the development of contextually applicable interventions and long‐term sustainability.^52^ The unique, multifaceted community engagement approach characterizing the present research allowed for the synthesis of perspectives from diverse rural community stakeholders and an in‐depth understanding of what community stakeholders find important and prioritize. The concept mapping methodology engaged stakeholders across the levels of the engagement spectrum.^53^ Because participants themselves were invited to collectively interpret the ideas generated (equitable and shared decision‐making), the results should have high translational and external validity.^54^ Additionally, the iterative concept mapping methodology itself facilitated collective brainstorming as stakeholders confronted and integrated different viewpoints, yielding innovative solutions that are greater than the sum of the knowledge and opinions of individuals.^27^, ^34^ Such a different approach from usual might have a lasting impact on participants' perception and engagement in community‐based research.

### 4.1 Limitations and suggestions for future research

Despite the strengths of the present research, there were also several limitations. The sample was composed of 8 (23.5%) adults under 45 years of age, 17 (50%) adults aged 45–65 and 9 (26.5%) participants were 65+ years. The majority were highly educated, identified with policy/government and education sectors, were very knowledgeable about digital technologies and had adequate internet access. The perspectives of other populations (e.g., younger, without internet access and/or less technology knowledge) should be explored in future research; in particular, because lack of internet access may have excluded people in remote areas inadvertently, including more participants living in very remote locations in future concept mapping research may provide unique perspectives on technological solutions for health equity. Our sample had a slightly higher representation of married and highly educated adults compared to Statistics Canada data for the regional districts comprising our target communities.^55^, ^56^, ^57^ Further, a smaller number of participants contributed to the sorting, rating and discussion of the final ideas generated, and although the demographic characteristics were similar to the overall sample, there was a high proportion with University degrees, suggesting there may have been over‐representation of participants with high levels of digital literacy in our sample. Likewise, ratings of feasibility could vary widely depending on stakeholders' level of expertise. Indeed, some of our participants expressed that without available support (e.g., funding, capacity) some solutions would not be as feasible. In the present study, the concept mapping design required an important cognitive effort as well as participants who were knowledgeable about and had access to technology. A larger sample to appreciate possible gender, age group, location, issues relevant to Indigenous populations and various experiences with other marginalized populations (e.g., those living in poverty) would be needed to reduce that bias. In the future, concept mapping as a methodology could be adapted to include semistructured interviews after Step 2 and before Step 3. Finally, since concept mapping is also a learning process, individual follow‐up with participants could be included after the group session to see what participants learned from the activity and if and how their feedback could translate into ‘real world actions’.

## 5 CONCLUSIONS

Overall, community stakeholders identified that technology solutions alone are not enough to promote rural health equity, but these require advancing the technology infrastructure, multistakeholder collaboration, community‐oriented or rural‐centric training and programmes and permanent human and technical support to ensure successful adoption. The concept mapping process engaged diverse rural community stakeholders in the co‐creation of technology solutions for rural health equity. The inclusion of rural community stakeholders in all steps of the concept mapping process generated innovation and user‐driven solutions towards rural health equity.

## AUTHOR CONTRIBUTIONS

Pierre Rondier, Kathy L. Rush and Eric P. H. Li contributed to the conceptualization of the project, and all authors contributed to the study design. Cherisse L. Seaton oversaw the data collection and facilitated the virtual discussion. Cherisse L. Seaton prepared Figure 1. Pierre Rondier prepared Table 1 and Figure 2. Pierre Rondier and Cherisse L. Seaton in collaboration with Polygon Inc. completed the data analyses including the preparation of Figure 3. Cherisse L. Seaton led overall manuscript development and integration. All authors contributed to manuscript drafts and reviewed the final manuscript.

## CONFLICT OF INTEREST

The authors declare no conflict of interest.

## Data Availability

The data sets in this study are based on are stored on secure servers at UBC Okanagan. Anonymized data are available upon request from the corresponding author.
